# Virulence Factors in Hypervirulent *Klebsiella pneumoniae*

**DOI:** 10.3389/fmicb.2021.642484

**Published:** 2021-04-08

**Authors:** Jie Zhu, Tao Wang, Liang Chen, Hong Du

**Affiliations:** ^1^Department of Clinical Laboratory, The Second Affiliated Hospital of Soochow University, Suzhou, China; ^2^Hackensack Meridian Health Center for Discovery and Innovation, Nutley, NJ, United States; ^3^Department of Medical Sciences, Hackensack Meridian School of Medicine, Nutley, NJ, United States

**Keywords:** *Klebsiella pneumoniae*, hypermucoviscous, capsule, siderophores, virulence factors, hypervirulence

## Abstract

Hypervirulent *Klebsiella pneumoniae* (hvKP) has spread globally since first described in the Asian Pacific Rim. It is an invasive variant that differs from the classical *K. pneumoniae* (cKP), with hypermucoviscosity and hypervirulence, causing community-acquired infections, including pyogenic liver abscess, pneumonia, meningitis, and endophthalmitis. It utilizes a battery of virulence factors for survival and pathogenesis, such as capsule, siderophores, lipopolysaccharide, fimbriae, outer membrane proteins, and type 6 secretion system, of which the former two are dominant. This review summarizes these hvKP-associated virulence factors in order to understand its molecular pathogenesis and shed light on new strategies to improve the prevention, diagnosis, and treatment of hvKP-causing infection.

## Introduction

*Klebsiella pneumoniae* is a common opportunistic pathogen that frequently causes nosocomial infections, including pneumonia, meningitis, bloodstream and urinary tract infection (Togawa et al., 2015; Ku et al., 2017). In addition, *K. pneumoniae* has the potential to cause community associated infection, such as liver abscess, endophthalmitis, and meningitis, in healthy individuals (Russo and Marr, 2019). An unique case of *K. pneumoniae* caused liver abscess along with endophthalmitis was reported for the first time in Taiwan in the 1980s, and the causative organism was designated as hypervirulent *K. pneumoniae* (hvKP) (Liu et al., 1986). Since then, hvKP has been recognized as another circulating pathotype in addition to classical *K. pneumoniae* (cKP), associated with high pathogenicity and mortality due to hypervirulence (Lan et al., 2020). Factors contributing to the hypervirulence mainly include capsule, siderophores, lipopolysaccharide (LPS) and fimbriae (Parrott et al., 2020).

The primary differences between cKP and hvKP are summarized in Table 1. The string test based on hypermucoviscous phenotype (string ≥5 mm) was widely used as a marker for hvKp, with ∼90% predicted accuracy for clinical hvKp strains (Russo et al., 2018). This semi-qualitative assay is easily influenced by colony conditions and the user’s technique (Tan et al., 2014). Also, cKP strains with mucoviscosity and hvKP strains without hypermucoviscosity have been identified (Catalán-Nájera et al., 2017). Another mucoviscosity assay was quantified by comparing the absorbance of supernatants collected by low-speed centrifugation because hypermucoviscous strains cannot sediment sufficiently, leaving the supernatants turbid (Walker and Miller, 2020).

**TABLE 1:** Main characteristic of cKP, hvKP, and MDR-hvKP.

**Characteristics**	**Classical *K. pneumoniae* (cKP)**	**Hypervirulent *K. pneumoniae* (hvKP)**	**Multidrug-resistant hvKP (MDR-hvKP)**	**References**
Infections	**Acquisition:** nosocomial **Host:** immunocompromised patients **Geographic region:** the whole world **Infectious sites:** urinary tract infections, pneumonia, bloodstream infections; usually polymicrobial at sites of infection **Metastasis:** uncommon	**Acquisition:** community **Host:** healthy adults **Geographic region:** Southeast Asia **Infectious sites:** pyogenic liver abscess, meningitis, endophthalmitis, necrotizing fasciitis; usually monomicrobial at sites of infection **Metastasis:** Common	**Acquisition:** nosocomial and community **Host:** usually immunocompromised patients **Geographic region:** Asia (especially China) **Infection sites:** pyogenic liver abscess, bloodstream infections, urinary tract infections	Harada et al., 2019; Russo and Marr, 2019; Liu C. et al., 2020; Tang et al., 2020
Phenotypes	Non-hypermucoviscosity and string <5 mm	Hypermucoviscosity and string ≥5 mm	Hypermucoviscosity or non-hypermucoviscosity	Russo et al., 2018
Common serotypes	K1–K79	K1, K2, K5, K16, K20, K54, K57, KN1	K1, K2, K16, K20, K54, K62, K64, K47	Pan et al., 2008, 2015; Yang et al., 2021
Siderophores	Enterobactin, yersiniabactin	Enterobactin, yersiniabactin, salmochelin, and aerobactin	Enterobactin, yersiniabactin, salmochelin, and aerobactin	Russo et al., 2015; Lam et al., 2018b; Choby et al., 2020

Except for intrinsic resistance to ampicillin, hvKp strains are commonly susceptible to a variety of antibiotics, including cephalosporins and carbapenems; however, multidrug-resistant and hypervirulent (MDR-hv) strains have recently emerged, primarily due to horizontal transfer of plasmid-mediated resistance or virulence (Liu C. et al., 2020; Tang et al., 2020). The spread of hvKP, including MDR-hvKP strains, poses a serious threat to the public health, and studies on hvKP virulence are therefore imperative to comprehend the underlying pathogenesis and provide a theoretical basis for the treatment and prevention of infection. In this review, we discuss hvKP relevant virulence elements, with reference to the discovery and epidemiology.

## Discovery and Epidemiology

In 1986, a striking case of *K. pneumoniae* liver abscess (KLA) with extrahepatic complications such as purulent meningitis, prostate abscess, and pyogenic ophthalmia, was recorded in Taiwan, wherein patients eventually went blind despite receiving active treatment for the bacteria (Liu et al., 1986). Historically, pyogenic liver abscess was regarded as a polymicrobial infection and its predominant bacterial cause was *Escherichia coli* (Wang et al., 1998; Lederman and Crum, 2005). A single microorganism *K. pneumoniae* caused liver abscess occurred in this case, which was alarming as new invasive syndrome. Coincidentally, several studies by Nassif and Sansonetti (1986) and Nassif et al. (1989a;b) reported the existence of a ∼180-kbp plasmid in *K. pneumoniae* strains, contained the genes encoding aerobactin and its receptor, as well as *rmpA* conferring a mucoid phenotype. A comprehensive hvKp definition refers to hypermucoviscous phenotype, the corresponding virulence gene genotype and clinical manifestation of metastatic infection (Catalán-Nájera et al., 2017). For some variants with ambiguous pathogenic potential, murine but not *Galleria mellonella* models are more appropriate to evaluate the hypervirulence phenotype accurately (Russo and MacDonald, 2020), although the *G. mellonella* lethality assay combined with the string test has been demonstrated to improve clinical hvKP identification significantly (Li et al., 2020).

Due to increasing population mobility, hvKP infections have spread worldwide, throughout Asia (Chung et al., 2007; Shankar et al., 2018), Europe (Moore et al., 2013; Cubero et al., 2016; Rossi et al., 2018; Sánchez-López et al., 2019), Oceania (Foo et al., 2018; Sturm et al., 2018), North-America (Rahimian et al., 2004; Pastagia and Arumugam, 2008; Fazili et al., 2016), South-America (Coutinho et al., 2014), and Africa (Yu et al., 2007), with the highest prevalence in the Asian Pacific Rim. Genetic factors and geographic conditions are likely to be relative risk factors for this situation (Sellick and Russo, 2018). The geographical distribution of MDR-hvKP also showed similar pattern that they were mostly found in Asian countries, although global spread has been documented (Li et al., 2014; Tang et al., 2020). The genetic backgrounds of hvKP varied, among which clonal group (CG) 23 strains are the most predominant (Liao et al., 2014; Shi et al., 2018). However, novel hvKp sequence types continue to emerge, indicated the ongoing evolution of hvKP (Shankar et al., 2018). MDR-hvKP strains mainly belonged to ST11, and carbapenem-resistant *K. pneumoniae* (CR-KP) MDR-hvKp ST11 strains have been described in China (Gu et al., 2018; Tang et al., 2020). Both ST11 and ST258 belong the CG258 clone and ST11 is a single locus variant to ST258. Despite the high prevalence of MDR-hvKp in ST11 strains, ST258 MDR-hvKP strains have not yet been detected (Ernst et al., 2020).

An epidemic of infectious diseases requires three indispensable segments: source, route, and susceptible population. In terms of habitat associations, *K. pneumoniae* have been frequently found in human, animal, sewage, polluted water, and soil samples (Bagley, 1985; Podschun et al., 2001); however, the origin of hvKP is yet to be ascertained. Liu et al. (2018) recently found ST23 hvKP in a cucumber sample along with *E. coli* carrying *bla*_NDM_ when analyzing ready-to-eat vegetables. In addition, three MDR-hvKP strains have been obtained from public aquatic environments in Brazil, suggesting the contaminated water source may potentially increase the risk of hvKP spread (Furlan et al., 2020). These results suggested that environment may serve as a reservoir for the dissemination of hvKp and MDR-hvKP strains.

*Klebsiella pneumoniae* isolates require suitable areas for colonization before setting off infections, and the gastrointestinal tracts are regarded as major reservoirs for *K. pneumoniae* colonization (Gorrie et al., 2017; Sun et al., 2019). A previous study investigated *K. pneumoniae* serotypes of stool specimens collected from healthy Chinese in Asian countries. The data presented that intestinal colonization rates of cKP and hvKP (K1/K2 serotypes) were 18.8–87.7 and 0–16.7%, respectively (Lin et al., 2012), while in Europe, the carriage rate of cKP carriers is 5.9–19.3% (Smith et al., 1997; Gorrie et al., 2017). Nevertheless, whether the difference in bacteria load among different worldwide regions could be ascribed to the incidence of highly invasive diseases caused by hvKP is yet to be confirmed. Additionally, Fung et al. (2012) discovered that serotypes and genotypes of bacterial isolates from healthy carriers’ fecal samples are identical to those of *K. pneumoniae* liver abscess (KLA) patients, which was in line with a family-clustered case report in Japan that the stool cultures from two healthy relatives of KLA patients were hvKP-positive, rendering them as carriers (Harada et al., 2011). Intestinal tract colonization by carbapenemase-producing *K. pneumoniae* has also been documented (Gorrie et al., 2017). For example, ST11 CR-hvKP strains have been isolated from stool samples of acute diarrhea inpatients in Zhejiang Province, China (Zheng et al., 2020). These findings indicated that guts might be a significant reservoir for hvKP strains as they can break the intestinal barrier and move to the liver to cause KLA and metastatic spread. Additionally, person-to-person transmission via a possible fecal-oral cycle is also possible (Siu et al., 2012), but the trigger for the occurrence of infection after colonization is not yet clarified.

Unlike cKP caused nosocomial infections, hvKP results in community-acquired infections (Ikeda et al., 2018; Harada et al., 2019). Once nosocomial cKP strains acquire hypervirulence plasmid and colonize in the intestine, healthcare-associated hvKP infections rise indiscriminately (Russo and Marr, 2019). A retrospective study conducted in Beijing by Liu C. et al. (2020) found that >90% hvKP-causing infections are nosocomial, suggesting the likelihood of hvKP evolved as a healthcare-associated pathogen. Retrospective studies showed hvKP infections mainly occur in susceptible individuals, male and aged over 50-years-old, and with an underlying condition of diabetes among (Fazili et al., 2016). Jin et al. (2020) reported that the bactericidal capacity of diabetic neutrophil extracellular traps (NETs) to hvKP was impaired, which partly explained why diabetics were vulnerable to hvKP infections. Another potential risk factor, co-existing hepatobiliary disease, was identified in a recent study (Parrott et al., 2020). In-depth studies analyzing the host factors to hvKP infections remain limited, while this knowledge could significantly contribute in unraveling the underlying epidemiology and implement appropriate prevention and control measures.

## Virulence-Associated Factors

### Capsule

The correlation between hypermucoviscosity and hypervirulence is widely accepted, albeit with a few exceptions. The capsule surrounding the surface of *K. pneumoniae* serves as main virulence factors associated with the viscous phenotype. The characterization of an intranasal infection model revealed that mice infected with acapsular strains of *K. pneumoniae* had longer survival significantly and lower bacterial density in lung and blood than those infected with capsular strains, as the capsule might provide protection from the immunological responses (Yoshida et al., 2000; Lawlor et al., 2005). Capsule-mediated resistance to bactericidal actions is inclined to be defensive rather than offensive. For example, *K. pneumoniae* evades phagocytosis, complement, antimicrobial peptides, and specific antibodies using capsules to make it difficult for bacteria to be bound, while active suppression and attack on immune cells through capsule are rarely observed (Domenico et al., 1994; Alvarez et al., 2000; Llobet et al., 2011; Paczosa and Mecsas, 2016). Furthermore, the capsule size of hvKP strains is above the basal level of cKP, and hvKP isolated from the cerebrospinal fluid is highly prevalent in a mean capsule size of >2 mm (Ku et al., 2017). Thus, these isolates have robust coats expressing hypermucoviscous phenotype that might enhance their viability. On the other side, the thick hypercapsule could serve as a physical barrier and impair the DNA uptake, consequently, limiting the horizontal gene transfer, which may partially explain why hvKp strains are less likely to harbor antimicrobial resistant genes than those of cKp strains (Wyres et al., 2019).

Based on the diversity of the polysaccharide components of the capsule and different structures and antigens, *K. pneumoniae* can be divided into at least 79 serotypes (Pan et al., 2015). Eight types, K1, K2, K5, K16, K20, K54, K57, and KN1 have been described in hvKP (Pan et al., 2008; Lee et al., 2016); with K1 and K2 the most common. Multilocus sequence typing (MLST) showed that the diversity of sequence-type (ST) was greater in capsule type K2 than K1 serotype that K1 was strongly related to ST23 while K2 was associated with ST25, ST86, ST375, and ST380, respectively (Struve et al., 2015; Lee et al., 2016). The prevalent serotypes differ with the geographic regions: K1 is the most predominant type in Asia, while K2 is more frequent found in Europe and North America (Catalán-Nájera et al., 2017; Remya et al., 2018). For MDR-hvKp, serotypes also depend on ST and region, as an example, serotypes K64 and K47, associated with the ST11 strains, are most common in China (Yang et al., 2021).

Several studies have suggested that K1 and K2 strains have higher virulence than the strains of other serotypes (Yeh et al., 2007; Wang et al., 2017), and hence, serotype is considered as one of the potential virulence factors, which could be attributed to the distinct compositions that confer survival advantages. A previous study showed that K1 and K2 capsule types lacked mannose and rhamnose, which could be recognized by macrophage lectin receptors to induce phagocytosis (Athamna et al., 1991; Fung et al., 2002). In addition, a surface-exposed protein, fructose-1, 6-bisphosphate aldolase (FBA), was identified in K1 hvKP, and its enhanced expression could protect bacteria against neutrophil killing under high glucose condition (Lee et al., 2020). Moreover, sialic acid, as a constituent of K1 and K2 capsular polysaccharide, contributes to hypermucoviscous phenotype, and is thus responsible for the anti-phagocytic activity directly or indirectly (Lee et al., 2014; Opal, 2014). These reports do not indicate equal hypervirulence among these serotypes, and K1/K2 cKP strains without increased viscosity and additional virulence factors usually fail to express hypervirulence phenotype. However, serotyping reports under the diagnosis of pyogenic liver abscess are valuable and could guide to take precautions to prevent further episodes (Fung et al., 2002).

**FIGURE 1:**
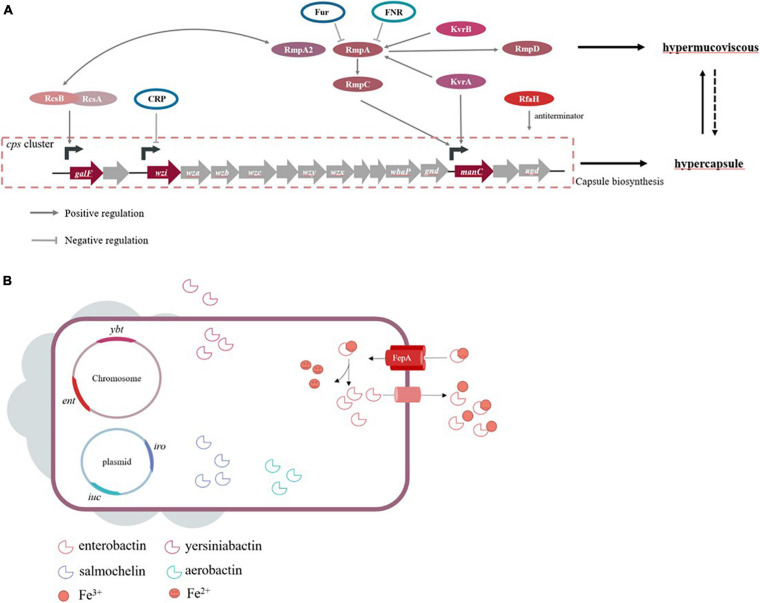
**(A)** Genes are known to influence capsule biosynthesis, including genes at *cps* locus and regulators affecting the *cps* expression directly or indirectly. The corresponding promoters that some regulators act on refer to studies of Walker and Miller (2020). Hypercapsule is sufficient but not necessary for hypermucoviscous phenotype, demonstrating that hypermucoviscous phenotype is not solely due to excessive capsule expression. **(B)** The siderophore systems enterobactin *(ent)*, yersiniabactin *(ybt)*, salmochelin *(iro)* and aerobactin *(iuc)* are present. Take enterobactin as an example to show the process of iron acquisition, which FepA is the transporter specific to enterobactin.

Genes involving capsule production are located on the capsular polysaccharide synthesis (*cps*) region of chromosome. The region of *cps* cluster (from *galF* to *ugd*) harbors over 20 genes, largely driving by three promotors located upstream of genes, *galF, wzi*, and *manC*, respectively (Figure 1A). Among these genes, *wbaP* encoded protein mediated the first step of capsule biosynthesis, and the deletion of this gene confers impaired capsule production. Interestingly, a recent study found that certain *wbaP*-mutations enhanced the pathogenicity by improving biofilm formation and bladder epithelial cells invasion in urinary tract infections (Ernst et al., 2020). The function of *wzx* in capsule-biosynthesis is flipping the polymer from the cytoplasm to the periplasm of the inner membrane. The Wzy protein is involved in the polymerization via catch-and-release mechanism (Islam and Lam, 2014). Whilst proteins encoded by *wza* and *wzc* genes form a translocation complex responsible for assembling capsular polysaccharides and transporting them from the periplasm to the surface of the bacteria (Pan et al., 2013). The Wzb protein, as the cognate phosphatase of Wzc, combines with the catalytic domain on Wzc, and in turn dephosphorylates Wzc (Reid and Whitfield, 2005; Niu et al., 2020). A study concluded that *wza* knockout strains obstruct the polysaccharide transport, decrease the expression of *wzb, wzc*, and *wzi* genes to avoid overaccumulation of polysaccharides in the cells, and ultimately lead to capsule deficiency (Niu et al., 2020). The serotype-specific gene *wzi* encodes an outer membrane protein that assists in the formation of the bacterial capsule by anchoring the capsular polysaccharides to the outer membrane, and *wzi*-deficiency confers acapsular phenotype (Rahn et al., 2003; Brisse et al., 2013; Bushell et al., 2013). The *wzy* gene is regarded as a serotype-specific target which has been frequently used as a target to characterize different *cps* types. Intriguingly, the chromosomal mucoviscosity-associated gene A (*magA*) discovered in 2004 was later found to be the *wzy* gene in K1 locus (i.e., K1_wzy) (Fang et al., 2004, 2010; Chuang et al., 2006). In addition, *magA* (K1_wzy) is essential for the synthesis of capsular polysaccharide and plays an important role in hypermucoviscous and resistance to complement-mediated lysis, while Δ*magA* mutants lose these features (Wu et al., 2009). Therefore, it is postulated that *magA* acts as a virulence-associated factor but is restricted to K1 isolates.

High capsule productivity is also attributed to a number of virulence genes, in addition to those present in the *cps* cluster, including the regulation of the capsule synthesis B genes (*rcsB*), a regulator of mucoid phenotype A and A2 (*rmpA* and *rmpA2*) and Klebsiella virulence regulators (*kvrA* and *kvrB*) (Cheng et al., 2010; Palacios et al., 2018; Su et al., 2018). These genes are positive transcriptional regulation factors for the *cps* locus. The Rcs phosphorelay system is a two-component signal transduction system, which was originally identified as a regulator for the synthesis of colonic acid in *E. coli* and composed of three core proteins, RcsC, RcsD, and RcsB (Wall et al., 2018). RcsC protein is a transmembrane sensor kinase that can undertake autophosphorylation after sensing extra-cytoplasmic stimulus and then transfer the stimuli to RcsD. Finally, the signal is transmitted to RcsB, which is the response regulator forming homodimer or heterodimer with the auxiliary proteins. Subsequently, the phosphorylated RcsB interacts with RcsA, and this dimer binds to promoters in the *cps* cluster, which activates the biosynthesis of the capsule (Guo and Sun, 2017). This mechanism of regulation in *E. coli* is applicable to other Gram-negative bacteria, but capsular polysaccharide-related genes controlled by auxiliary regulator RcsA are limited in comparison to RcsB in *K. pneumoniae* (Su et al., 2018). On the other hand, the *rmpA* gene of *K. pneumoniae* expresses *rcsA*-like activity and complements a *rcsA* mutant of *E. coli* to positively control the mucoid phenotype (Nassif et al., 1989b; Arakawa et al., 1991; Gottesman and Stout, 1991). Similar to RcsA, RmpA depends on RcsB for activating the biosynthesis of capsular polysaccharides. Together, *rmpA*^+^*rcsB*^–^ strains and *rmpA*^–^*rcsB*^+^ strains are unable to increase capsule production (Cheng et al., 2010). Consistent with this theory, RcsB seems vital for base-level capsule expression, while the regulator RmpA elevates the capsule production (Walker et al., 2019). Moreover, partial homology was also detected in *rmpA2*, *rmpA*, and *rcsA* (Wacharotayankun et al., 1993). While RmpA2 is directly bound to the *cps* gene promoter to regulate capsule productivity, *rcsB*^–^ *K. pneumoniae* strain carrying *rmpA2* recovers the mucoid phenotype (Lai et al., 2003). Furthermore, RmpA and RmpA2 are known regulators required for hypermucoviscous phenotype in hvKP strains. Different from *rcsA*, *rcsB*, and *rcsC* that were found in chromosome, both *rmpA* and *rmpA2* could be carried by the plasmid or chromosome, wherein chromosomal *rmpA* (c-*rmpA*) was located in an integrative and conjugative element (ICE*Kp1*), and plasmidic *rmpA* (p-*rmpA*) was most prevalent. Hsu et al. (2011) found that the K1 strain NTUH-K2044 contained three *rmpA/rmpA2* (p-*rmpA*, p-*rmpA2*, and c-*rmpA*), only p-*rmpA* could enhance the expression of *cps* genes, while p-*rmpA2* and c-*rmpA* had inhibitory and little effects, respectively. Notably, not all *rmpA/rmpA2* genes can upregulate the capsule expression, and hence, *rmpA/rmpA2* genes in *K. pneumoniae* strains lacking a hypermucoviscous phenotype are also isolated. Yu et al. (2015a;b) showed that p-*rmpA/rmpA2* could occur in concurrence to frame-shift mutations in absence of c-*rmpA*. In addition, the process of capsule production needs an important transcription factor, RfaH, which actives the *cps* operon by inhibiting transcription termination (Bachman et al., 2015; Svetlov et al., 2018). The newly described regulators, *kvrA* and *kvrB*, affect virulence of K1/K2 hvKP strains due to the activation of capsule gene expression, which is not present on cKP strains. Also, the impact of KvrB on capsule expression may be dependent on its effect at the *rmpA* promoter in contrast to that of KvrA (Palacios et al., 2018; Walker and Miller, 2020).

The factors that influence capsule production involve various environmental stimuli in addition to the elements mentioned above. *K. pneumoniae* strains are facultative anaerobes growing under aerobic or anaerobic conditions, and hence, oxygen availability is crucial for their survival. Fumarate nitrate reduction regulator (FNR), an oxygen-responsive transcriptional regulator, contains a [4Fe-4S] cluster in the N-terminal sensory domain regulating the C-terminal DNA binding domain. FNR can be activated via the [4Fe-4S] cluster binding to DNA, specifically during anaerobic environment, while the [4Fe-4S] cluster is oxidized, causing the loss of FNR activity under aerobic conditions. Lin et al. (2019) found that activated FNR inhibits the transcription of *rmpA/rmpA2* and decreases the amount of capsular polysaccharide during anaerobic growth. Capsular polysaccharide biosynthesis is also related to environmental iron availability. Some studies reported that the ferric uptake regulator Fur participates in the repression of RmpA, RmpA2, and RcsA that are responsible for capsule production (Lin et al., 2011). Moreover, Fur directly inhibits the transcription of non-coding RNA RyhB via binding to its promoter region, which reduces the amount of capsule independent of RmpA, RmpA2, and RcsA (Huang et al., 2012). The functional role of Fur entails regulated production of siderophores and capsules. cAMP receptor protein (CRP) decreases the expression of *cps*, which is a consequence of direct interaction between *manC* and *wzi* promoters (Ou et al., 2017). In response to exogenous glucose, *K. pneumoniae* produces capsular polysaccharide via cAMP-dependent carbon catabolite repression, which might be a probable cause of high KLA prevalence among diabetic patients (Lin et al., 2013).

The regulation of most genes mentioned is aimed at the *cps* cluster to affect capsule synthesis; however, the correlation between hypermucoviscosity of hvKP and capsule expression has not yet been clarified. Recently, Walker et al. (2019, 2020) identified two new genes (*rmpC* and *rmpD*) neighboring *rmpA* on the virulence plasmid. The Δ*rmpC* maintained the downregulated expression of capsule genes but preserved hypermucoviscosity, while *rmpD* mutants were hypermucoviscosity-negative and had no change in capsule production. As *rmpA-*deficient stains showed reduced *cps* expression and the loss of hypermucoviscosity phenotype, the author speculated that expressions of *rmpD* and *rmpC* are both driven by RmpA but regulate hypermucoviscosity phenotype and *cps* expression separately (Walker et al., 2019, 2020). Strikingly, hypercapsule is not a sole cause of hypermucoviscosity phenotype, and early reports have already proposed that the generation process of hypercapsule and hypermucoviscosity is different (Nassif et al., 1989a,b). The hypermucoviscosity phenotype might be attributed to the heterogeneity of capsule composition in addition to thick capsules, which described that KLA isolations contain fucose synthesized by genes *wcaG* and *gmd*, while the absence of these genes was verified in the classical strains of *K. pneumoniae* causing urinary tract infection (Wu et al., 2008). Pan et al. (2011) made a further exploration via disrupting the fucose synthesis gene in this KLA isolate; the results showed that the mutant displayed less mucoid than the wild-type, but the capsules were still visible. Briefly, hypercapsule is an indispensable virulence factor, but insufficient; either unknown functions of reported genes or additional regulatory factors could be the possible causes of hypermucoviscosity that require further exploration.

### Siderophore

For hosts and bacteria, the metal iron is a critical element required for essential metabolic processes, and the limited availability of iron within extracellular fluid poses a difficulty for bacterial iron acquisition, rendering it a form of non-specific immune defense. If bacteria intend to survive and grow, they need to employ tactics to overcome this difficulty of iron acquisition. In human hosts, the iron ingestion is generally in need of a siderophore-dependent iron acquisition system as the result of the insolubility of free iron Fe^3+^ during physiological conditions. Similarly, the majority of the bacteria, including *K. pneumoniae*, possess this system with a higher affinity for iron than the host, which serves as a predominant strategy of acquiring iron in order to survive the competition with hosts (Miethke and Marahiel, 2007), while another solution employed by only a minority of pathogens, such as *Borrelia burgdorferi* and *Treponema pallidum*, is avoiding the need for iron by eliminating genes encoding iron-dependent proteins (Posey and Gherardini, 2000). Siderophores are small iron-binding molecules that are synthesized inside the bacteria and secreted outside the cells, following which, they bind to environmental iron and transport it back to the cells. Subsequently, iron-siderophore complexes are recognized by the specific outer-membrane receptors transporting the corresponding material to the periplasm, where siderophores combine with the periplasm proteins to transport them to the inner membrane. Finally, iron passes through the passage mediated by an ABC-transporter into the bacterial cytoplasm, where the ferric iron is reduced into ferrous iron accessible to bacteria (Brown and Holden, 2002; Khan et al., 2018). Previous studies by Rogers (1973) described that iron-binding chelators were considered as the true virulence factors in *E. coli* owing to their key roles in rapid bacterial growth. This hypothesis is commonly accepted and applicable to other pathogens. Previous studies have shown that hvKP presents a distinguishing 6- to 10-fold increase in siderophore production as compared to cKP (Russo et al., 2014). Four siderophores are expressed in hvKP strains, including enterobactin, yersiniabactin, salmochelin, and aerobactin; these improve the growth efficiency of the bacteria.

Among four siderophores, the conserved enterobactin contains three catechol rings that confer the highest binding affinity for Fe^3+^, which renders them essential and primary for both cKP and hvKP in iron uptake (Bachman et al., 2012; Palmer and Skaar, 2016). The genes required for enterobactin biosynthesis constitute the *ent* cluster localized on the chromosome, while its corresponding receptor is encoded by *fepA* (Baghal et al., 2010) (Figure 1B). In response, hosts produce the siderophore-binding protein lipocalin-2 that have antimicrobial capabilities by hampering iron acquisition rather than killing pathogens directly (Holmes et al., 2005). *K. pneumoniae* strains producing only enterobactin will cause them to fail owing to the presence of lipocalin-2 (Bachman et al., 2011). Smartly, a subset of *K. pneumoniae* strains evolves stealth siderophores to evade lipocalin-2 binding, such as the highly glycosylated enterobactin, salmochelin, and the alternative non-catecholate siderophore, yersiniabactin. In other words, the strains with salmochelin and yersiniabactin acquire iron to survive despite the presence of linpocalin-2 (Bachman et al., 2012; Holden et al., 2014). The synthesis, excretion, and uptake of salmochelin are carried out by genes of the *iroA* locus, *iroBCDN*, among which IroN and IroB are similar to the enterobactin receptor FepA and glycosyltransferases, respectively (Lin et al., 2005; Müller et al., 2009). In addition, a chromosomal copy of *iro* (ICE*Kp1*) is detected in the DNA fragment in the strain NTUH-K2044 (Lin et al., 2008). Yersiniabactin, a part of *Yersinia* high-pathogenicity island (HPI) present in chromosomal structures ICEs, is synthesized by the *ybt* locus (Bach et al., 2000). ICEs are mobile genetic elements (MGEs) transmitting these virulence determinants within the bacterial populations via horizontal gene transfer. Lam et al. (2018a) analyzed 2,498 genomes and found that *ybt* loci were divided into 17 distinct lineages and associated with 14 distinct ICE*Kp* structures (ICE*Kp1* ∼ ICE*Kp14*). In addition to ICE*Kp1* of NTUH-K2044, the most common ICEs in CG23 hvKP appear to be ICE*Kp10*, resembling the genomic island KPHPI208 in liver abscess strain 1084. A unique region located in this element is the synthesis locus of colibactin and microcin E492, which may play a role in the pathogenicity of hvKP (Struve et al., 2015). Colibactin is necessary for the full virulence of strain 1084 to develop meningitis, and microcin E492 presents its antibacterial capacity during gastrointestinal colonization by attaching to salmochelin (Lagos et al., 2001; Lu et al., 2017). Recently, a novel ICE variant named ICE*KpSL1* with high homology to *Yersinia* HPI was detected in a hvKP strain SCsl1 isolated from the liver of infected pigs (Liu D. et al., 2019). Typically, all the four siderophore determinants can be plasmid-borne and potentially transferable. As an example, the FIB_k_ plasmid-borne *ybt* is identified as a novel mechanism for *ybt* dispersal (Lam et al., 2018a).

In an hvKP background, the data shows that the proportion of aerobactin in the total siderophore production is about 90% and that it has the predominant contribution to hypervirulence under experimental conditions (Russo et al., 2014, 2015). A previous study demonstrated that hvKP mutants with aerobactin deficience, but not enterobactin, salmochelin, and yersiniabactin, decreased virulence in mice after pneumonic and subcutaneous infection (Russo et al., 2014, 2015). Aerobactin is expressed in >90% hvKP strains but only in 6% cKP strains, and aerobactin is more specific for hvKP as compared to enterobactin and yersiniabactin, suggesting it may be a reliable biomarker for hvKP (Paczosa and Mecsas, 2016; Russo et al., 2018). Similarly, aerobactin is more sensitive in defining hvKP than hypermucoviscosity (Zhang et al., 2016; Lin et al., 2020). The genes responsible for aerobactin biosynthesis and transport, *iucABCD* and *iutA*, are found on the virulence plasmid (Bailey et al., 2018; Russo and Gulick, 2019). The *iuc* locus structure in *K. pneumoniae* presented genetic diversity, resolving into six *iuc* lineages. Most of these lineages were mobilized by diverse plasmids, with the exception of *iuc4* that was localized on the chromosome of *K. pneumoniae* subspecies *rhinoscleromatis* (Lam et al., 2018b). Interestingly, sequence analysis revealed that pLVPK, a plasmid initially described in the K2 strain *K. pneumoniae* CG43, simultaneously contains genetic loci *iucABCDiutA* and *iroBCDN*, along with *rmpA* encoding hypermucoviscous phenotype, which is uncommon in cKP strains (Chen et al., 2004; Tang et al., 2010; Liu and Guo, 2019). Yu et al. (2007) found that aerobactin producer was observed in 96% of *rmpA* gene-positive isolates after analyzing 455 *K. pneumoniae* bacteremia in seven countries. The phenomenon that aerobactin production is concomitant with the mucoid phenotype might imply the potential correlation between areobaction and RmpA, but needs to be substantiated with additional studies.

The analysis of the genomic sequence of hvKP strain NTUH-K2044 identified an additional iron transport system, *kfu*, localized on the chromosome, which is also prevalent in hvKP as compared to the cKP strains. It is also regarded as a clone-specific marker because it is not conserved in other clones except CG23 (Wyres et al., 2020). Kfu was shown to be a potential virulence factor, and murine experiments demonstrated that Δ*kfuABC* mutants presented lower virulence than wild-type strain (Ma et al., 2005). Interestingly, this effect only occurs in mice infected intragastrically with the *kfu* mutant, but not in intraperitoneal infection, which indicates that *kfu* might contribute to intestinal colonization. Furthermore, the discrepancy among *iroA*, *Yersinia* HPI, *iucABCDiutA*, and *kfu* is that the first three systems are TonB-dependent (Hsieh et al., 2008). Based on of these findings, the presence of these multiple and redundant iron transport systems is reasonable and complementary because they can function in different organs or microenvironmental conditions during infection.

Although iron is necessary for bacterial growth, based on the Fenton reaction, superfluous Fe^2+^ ions catalyze the generation of hydroxyl radicals, i.e., reactive oxygen species (ROS) oxidizing lipids and macromolecules, thereby damaging the cells (Lloyd et al., 1997). The above-mentioned Fur regulates the expression of *rmpA* and siderophore synthetic gene expression. Under iron-rich conditions, Fur binding to Fe^2+^ represses siderophore synthesis by affecting the Fur box in the promoters of genes related to iron uptake, thereby avoiding iron overload (Hahn et al., 2000). For example, in *K. pneumoniae* CG43, Fur could bind to the Fur box sequences in the coding region of *iucA*, *iroB*, and *entC* to play its regulatory role, as described by Lin et al. (2011). The promoter sequences of *kfu* operon also contain the conserved Fur box (Ma et al., 2005). Emerging evidence supports that Fur acts as a transcriptional activator, increasing the expression of active enzymes against toxic ROS. Thus, Fur contributes to virulence, and *fur* mutants exhibit a decrease in virulence and inability to cause disease within the animal host (Troxell and Hassan, 2013).

### Other Virulence Factors

Lipopolysaccharide consists of lipid A, oligosaccharide core, and O antigen and is known as the endotoxin of all Gram-negative bacteria, both cKP and hvKP. Presently, it is unclear whether LPS produced by hvKP strains has a unique role in hypervirulence. As the outermost subunit of LPS, O-antigen is the first molecule to be encountered by the host’s innate immune system and protect the pathogens against complement-mediated killing. Specifically, the O antigen binds a complement component C3b involving pore-formation to prevent drilling of the bacterial membrane (Shankar-Sinha et al., 2004). The number of O serotypes is estimated to be eight, and O1 antigen is the most common among clinical *K. pneumoniae* strains (Hansen et al., 1999; Follador et al., 2016). Previous studies have shown that O-antigen in O1:K2 cKP might contribute to bacterial virulence and lethality by weakening macrophage activation and promoting bacteremia (Lugo et al., 2007). Hsieh et al. (2012) demonstrated that the prevalence of O1 serotype in KLA isolates is higher than that in non-tissue-invasive isolates, and KLA strains with defect O1 antigen were less virulent than parental strains. However, its function has not yet been embodied in K1 hvKP strains, in which O1 antigen is masked by K1 capsule (Merino et al., 1992; Hsieh et al., 2012).

The types of fimbriae characterized experimentally for *K. pneumoniae* are type 1 and type 3 fimbriae. In the genome of hvKP NTUH-2044, seven novel fimbrial gene clusters, *kpa*, *kpb*, *kpc*, *kpd*, *kpe*, *kpf*, and *kfg*, were identified, and the Kpc fimbriae are highly associated with K1 serotype hvKP (Wu et al., 2010). Type 3 fimbriae constitute well-known bacterial virulence factors, mediating enhanced biofilm formation on abiotic surfaces, however, only little is known about their role in hvKP strains (Schroll et al., 2010). Using *K. pneumoniae* CG43, Wu et al. (2012) proved that type 3 fimbriae activity and biofilm formation could be influenced by iron availability and positively controlled by Fur, suggesting that type 3 fimbriae might be less important for hvKP infection *in vivo* under the condition of limited iron (Shon et al., 2013). A recent study also reported that hypermucoviscous isolates with serotype K1, despite harboring *mrkD* and *fimH* genes encoding type 3 and type 1 fimbriae, respectively, presented low initial adhesion because the presence of hypercapsule could hide these fimbriae (Cubero et al., 2019). Another adhesive structure, a non-fimbrial protein CF29K, is prevalent in hvKP strains, indicating its involvement in KLA pathogenesis or association with other pathogenicity factors in the same plasmid (Darfeuille-Michaud et al., 1992; Brisse et al., 2009).

Several outer membrane proteins (OMPs), including OmpA, peptidoglycan-associated lipoprotein (Pal), and murein lipoprotein (LppA), contribute to *K. pneumoniae* virulence. In response to intraperitoneal infection with *ompA*-, *pal*-, and *lppA*-deficient NTUH-20444, only the *pal* and *lppA* mutants yielded high LD50 values (50% lethal dose) than wild type strains, meaning lower virulence. The mechanisms behind the decreased virulence were likely to be that these mutants were easily killed by serum and neutrophil. Remarkably, *pal* and *lppA* mutants with low virulence retained intact K1 and O1 antigen inducing antibodies production and mice immunization, which indicated that these mutant strains could be meaningful for vaccine development (Hsieh et al., 2013). Similarly, *K. pneumoniae* also produces two major porins, OmpK35 and OmpK36. The downregulation of these molecules could be ascribed to antibiotic resistance and virulence sacrifice of the bacteria. In a mouse peritonitis model, the loss of both *ompK35* and *ompK36* in KLA isolate NVT2001 significantly reduced the pathogenicity and growth rate, thereby leading its elimination from the immune system (Tsai et al., 2011). This phenomenon is consistent with a previous report that disruption of *ompK36* failed to colonize the liver persistently due to the rapid clearance of bacterial burden (Chen et al., 2010). Srinivasan et al. (2012) found a novel porin in *K. pneumoniae* NTUH-2044, termed as KpnO. With respect to virulence, Δ*kpnO* produced lesser capsular polysaccharide and killed *Caenorhabditis elegans* slowly than wild-type strains. However, mechanisms underlying the attenuated virulence due to absence of these OMPs are yet to be elucidated.

In recent years, a new factor associated with virulence, type 6 secretion system (T6SS), has been identified in hvKP strains by *in silico* analysis. T6SS functions as a nanosyringe that injects effector proteins into target cells to destroy eukaryotic cells or contend against other prokaryotic cells (Sarris et al., 2011). Although >25% Gram-negative bacteria encode T6SSs, wild-type NTUH-2044 has been confirmed to outcompete *Salmonella* Typhimurium, *E. coli*, and T6SS-null *K. pneumoniae* in a T6SS-dependent manner, and the frequency of T6SSs in KLA strains is significantly higher compared to that in the intestinal-colonizing strains, suggesting that T6SSs may confer an advantage for hvKP survival (Clemens et al., 2018; Hsieh et al., 2019). Another study reported that in bacterial competition, *K. pneumoniae* T6SSs were activated by the PhoPQ system, followed by the use of effectors to kill *E. coli* prey that lack T6SS immunity genes (Storey et al., 2020). Moreover, the overexpression of a T6SS effector in the carbapenemase-producing *K. pneumoniae* strain, the phospholipase Tle1, was found to inhibit the growth of *E. coli* (Liu et al., 2017). Furthermore, in a hypervirulent strain Kp52.145, another T6SS effector gene *pld1*, encoding a phospholipase D family protein (PLD1), located within the T6SS locus, was reported as a virulence factor, exhibiting that Δ*pld1* was avirulent in comparison to wild-type in a mouse model of pneumonia (Lery et al., 2014). The previously sequenced genome showed that T6SS genes are clustered in two contiguous loci, while Barbosa and Lery (2019) later analyzed the neighboring genes and found that the genes coding for iron uptake systems is adjacent to T6SS-related genes: *tssD* in Kp52.145. Thus, it was hypothesized that T6SS is involved in iron import in hvKP (Barbosa and Lery, 2019).

## Convergence of Hypervirulence and Multidrug Resistance in *K. pneumoniae*

Increasing reports have described the emergence of multidrug-resistant hypervirulent *K. pneumoniae* strains (Hao et al., 2020; Shankar et al., 2020). MDR-hvKP can arise by two different mechanisms. In one case, hvKP strains can acquire antimicrobial resistance genes or plasmids through horizontal transfer, therefore becoming MDR-hvKP, which was tentatively designated as type I MDR-hvKP. For example, two carbapenemase plasmids, *bla*_NDM–__1_-bearing IncN plasmid and *bla*_KPC_-2-carrying IncFIIK plasmid, were recovered together from a K2 ST86 MDR-hvKP strain (Liu Y. et al., 2019). Additionally, MDR-hvKP strains can also arise from the transfer of a pLVPK-like virulence plasmid into a classic MDR *K. pneumoniae* strain, named as type II MDR-hvKP. As an example, a recent study in China described a fatal outbreak caused by a KPC-producing ST11 strain acquiring a pLVPK-like virulence plasmid (Gu et al., 2018). Type I MDR-hvKP strains share the same virulence determinants as the hvKP strains described above, while the hypervirulence in type II MDR-hvKP is mainly attributed to the over production of capsule and siderophore, as a result of the acquisition of plasmid-mediated virulence determinants, including *rmpA*/*rmpA2*, *iut* and *iro*. A recent study validated that five biomarkers (*peg344, iroB, iucA, prmpA*, and *prmpA2*), and ≥30 μg/mL siderophore production could accurately identify hvKP (Russo et al., 2018). The gene *peg-344* present on the virulence plasmid in hvKP including both type I and type II MDR-hvKP strains, had potential application in rapid diagnosis of hvKP, and a *peg-344* loop-mediated isothermal amplification technology had recently been established (Liao et al., 2020). PEG344 acted as a transporter in the inner membrane, but its specific role in virulence is unclear. Interestingly, Bulger et al. (2017) found that PEG344 was required for maximal virulence in pulmonary challenge model, but not in subcutaneous challenge.

## Summary

Following the early reports of hvKP in Asia, hvKP strains have spread globally, causing both community associated and hospital associated infections. The convergence of hypervirulence and antimicrobial resistance in hvKp further exacerbates this public crisis, and challenges the clinical treatment options for hvKp infections. Epidemiologically, the source and route of hvKp infection remain poorly understood, but intestinal colonization appears to be a critical step prior to infection, while susceptible factors include diabetes mellitus, male gender, and Asian ethnicity. Capsule and siderophores are predominant virulence factors that play a major role in the hypermucoviscosity phenotype of hvKP. However, molecular mechanisms under hypermucoviscosity remain to be explored as hypervirulent strains without hypermucoviscosity phenotype are frequently identified. The current definition of hvKP considers clinical characteristics, phenotypes, and genotypes, however, microbiologically determination of hvKp strains in clinical lavatories remains to be a challenge. Additional virulence factors include LPS, fimbriae, outer membrane proteins, and T6SS, whose functions in hvKP need to be further explored (Table 2). These factors are coordinated and regulated when bacteria orchestrate a series of events causing infection. Moreover, with the emergency of MDR-hvKP, the development of non-antimicrobial therapy targeting virulence factors is indispensable. Taken together, further studies on hvKp are needed to improve the prevention, diagnosis, and treatment of hvKP caused infections.

**TABLE 2:** Summary of virulence factors of hypervirulent *K. pneumoniae.*

**Category**	**Substance**	**Function**	**References**
Capsule	Wzi, Wza, Wzb, Wzc, Wzy, WbaP	Synthesize capsular polysaccharides	Islam and Lam, 2014; Ernst et al., 2020; Niu et al., 2020
	RcsB, RmpA, RmpA2, RmpC, RmpD, KvrA, KvrB, RfaH	Activate the biosynthesis of capsule	Palacios et al., 2018; Su et al., 2018; Svetlov et al., 2018; Walker and Miller, 2020
	FNR, Fur, CRP	Inhibit capsule production	Huang et al., 2012; Ou et al., 2017; Lin et al., 2019
Iron acquisition	Siderophores: Enterobactin, yersiniabactin, salmochelin, Aerobactin	Bind to environmental Fe^3+^ iron	Russo et al., 2015; Lam et al., 2018b; Choby et al., 2020
	ABC-transporter: Kfu	Transport Fe^3+^ into cytoplasm	Wyres et al., 2020
	Fur	Represses siderophores synthesis	Hahn et al., 2000
LPS	O-antigen	Avoid complement-mediated killing	Shankar-Sinha et al., 2004
Adhesion	Type 3 fimbriae	Important for biofilm formation on abiotic surface	Schroll et al., 2010
OMPs	PalA, LppA, OmpK35, OmpK36, KpnO	Protect against neutrophil phagocytosis	Tsai et al., 2011; Srinivasan et al., 2012; Hsieh et al., 2013
T6SS	PLD1, Tle1	Inject effector proteins into target cells, causing destruction	Lery et al., 2014; Liu et al., 2017; Storey et al., 2020

## Author Contributions

JZ drafted the manuscript. TW, LC, and HD revised the manuscript critically for important intellectual content. All authors contributed to the article and approved the submitted version.

## Conflict of Interest

The authors declare that the research was conducted in the absence of any commercial or financial relationships that could be construed as a potential conflict of interest.
